# Health Disparities: Statin Prescribing Patterns Among Patients with Diabetes in a Family Medicine Clinic

**DOI:** 10.1089/heq.2021.0144

**Published:** 2022-04-08

**Authors:** Fahamina Ahmed, Jonathan Lin, Taha Ahmed, Danish Siddiqui, John Nguyen, Daniel Sarpong

**Affiliations:** ^1^ Division of Clinical and Administrative Sciences, College of Pharmacy, Xavier University of Louisiana, New Orleans, Louisiana, USA.; ^2^ School of Medicine, Ross University, Miramar, Florida, USA.; ^3^ School of Medicine, American University of Integrative Sciences, Tucker, Georgia, USA.

**Keywords:** stain prescribing, health disparities, diabetes, ASCVD

## Abstract

**Purpose:**

To analyze the impact of gender and race on statin prescribing patterns in patients with diabetes in a family medicine clinic.

**Methods:**

This study (*n*=192) was a single-center, cross-sectional study that examined statin prescribing patterns at a family medicine clinic. Patients were obtained from January 2015 to November 2018, who were considered eligible for statin therapy based on a documented diagnosis of diabetes. The patients were divided into four subgroups for analysis (white males, non-white males, white females, and non-white females).

**Results:**

Females were found to have higher rates of prescribed statin therapy and appropriate statin intensity therapy when compared to males (*p*>0.05). When evaluating gender and race, white females were more likely to be prescribed an appropriate statin when compared to non-white females (*p*<0.05).

**Conclusion:**

The study shows that although males had a significantly higher mean 10-year atherosclerotic cardiovascular disease risk score, they were less likely than females to receive the appropriate intensity statin. Previous studies have shown race and gender disparities exist in the prevention of cardiovascular disease. A more collective, unified approach to improve prescribing patterns for statin therapy can eliminate these disparities.

## Introduction

Patients with diabetes have an increased lifetime risk of atherosclerotic cardiovascular disease (ASCVD).^1^ Statin therapy has proven to be one of the cornerstones in the primary prevention of cardiovascular disease, as well as for secondary prevention in patients who experienced a cardiovascular event.^2^ The 2018 ACC/AHA Guidelines state that the following groups would benefit from statin therapy: patients with a history of ASCVD, patients with a low-density lipoprotein (LDL) level ≥190 mg/dL, those 40 to 75 years of age with diabetes mellitus, and nondiabetic patients 40–75 years of age with a 10-year ASCVD risk ≥7.5%.

The 2018 ACC/AHA Guidelines recommends that patients with diabetes, 40 to 75 years of age, receive a moderate-intensity statin, while estimating risk factors to consider advancing to a high-intensity statin.^3^ Diabetic patients with a higher risk of cardiovascular disease may require statin intensification; however, risks versus benefits must be considered when moving up to a higher intensity statin due to any intolerable adverse event. A study conducted in patients with diabetes showed that statin intensification from low-dose to high-dose statin significantly decreased LDL levels; however, the amount of patients who discontinued statins due to adverse effects was also higher among those using a high-intensity statin.^4^

In patients who are eligible for statin therapy, males tend to receive a substantially higher frequency of statin treatment compared to their female counterparts.^5^ Although guidelines have highlighted the groups who could benefit from statin therapy, disparities continue to persist among females as well as African American patients.^6^ Some reasons for this disparity could stem from factors relating to the prescribing patterns, such as the belief that women have lower risks for cardiovascular disease or the underutilization of the available ASCVD risk estimating tools.^7^ Conversely, many contributing factors for the underutilization of statin therapy among female patients are patient specific, such as lower adherence rates and higher reports of adverse events compared to men.^8^

A former study conducted by Billimek et al. showed that in a cohort of 1,369 patients with diabetes, females were more likely to report adverse events due to statin use compared to males (47.2% vs. 37.8%, respectively, *p*=0.02), which ultimately may lead to the underuse of statin therapy among females.^9^ The Heart Protection Study was a landmark trial that studied the impact of statin treatment in patients with diabetes, which concluded that statin use was associated with a 22% reduction in coronary events and strokes.^10^ In the Collaborative Atorvastatin Diabetes Study (CARDS) trial, type 2 diabetic patients, 40 to 75 years of age, were randomized to receive a moderate-intensity statin or placebo. The results from the study showed that those randomized to receive atorvastatin 10 mg had a 37% relative risk reduction in having a major cardiovascular event.^11^

With multiple studies, such as the CARDS trial and Heart Protection Study, demonstrating a significant reduction in cardiovascular events in patients on statin therapy, it is essential that patients with diabetes receive a statin of at least moderate intensity regardless of their gender and race.^10,11^ A previous study conducted by Nanna et al. examined the differences in statin prescribing according to gender.^12^ The results of the study show that female patients who were less likely to be prescribed a statin were also less likely to be prescribed the optimal intensity statin when compared to males.^12^

The objective of this study is to analyze the impact of gender and race on statin prescribing patterns in patients with diabetes in a family medicine clinic. In addition to gender and race, other factors will also be assessed to determine any correlation with statin prescribing. Based on previous studies published in the *Journal of American Heart Association* (JAHA) regarding statin therapy, it is hypothesized that in our population, fewer female patients will be on adequate statin therapy compared to male patients.^13^

## Methods

This study was a single-center, cross-sectional study that examined statin prescribing patterns among diabetic patients at a family medicine clinic. The pharmacy team, which included a clinical pharmacist, a pharmacy resident, and pharmacy students, obtained a list of patients from January 2015 to November 2018, who were considered eligible for statin therapy based on the benefit group of interest documented diagnosis of diabetes.

Inclusion criteria for this study included patients who were considered eligible for statin therapy, defined as having a documented diagnosis of diabetes during a 47-month time period between January 2015 and November 2018. Exclusion criteria were patients without a documented diagnosis for diabetes, an LDL >190 mg/dL, or patients with an age <40 years. Patients with a diagnosis of clinical ASCVD were excluded as this population would be eligible for secondary prevention with statin therapy. Clinical ASCVD was defined as coronary heart disease (history of acute coronary syndrome, myocardial infarction or ischemia, stable or unstable angina, and/or coronary or other arterial revascularization) as well as peripheral arterial disease or stroke (including transient ischemic attack) of presumed atherosclerotic origin.

Additional exclusion criteria were pregnancy or breastfeeding, active liver disease, missing or incomplete data, and previously established contraindication, intolerance, or allergy to statins. Health conditions were identified from the electronic medical records using the International Classification of Diseases (ICD) codes.

A chart review was performed to determine the presence of gender and race disparities in statin prescribing, in addition to possible predictors of disproportionate statin prescribing. The following characteristics were obtained: age, race, gender, A1C level, systolic blood pressure, LDL, high-density lipoprotein (HDL), total cholesterol, smoking status, presence of hypertension treatment, presence of documented ASCVD risk, prescribed statin information, and any change in statin therapy. The specific prescribed statin, as well as the statin intensity, was recorded and compared to each patient's recommended statin intensity based on 2018 ACC/AHA Guidelines along with changes and reasons for statin therapy.^3^ Furthermore, ASCVD risk was calculated using all patients as the majority of patients did not have one previously documented. The research protocol was approved by the hospital's Institutional Review Board.

### Statistical analysis

Descriptive statistics was performed to describe the characteristics of the study sample. Means, standard deviations (SDs), and ranges were computed for continuous variables and frequency distribution (*n* and percentages) for categorical variables. Using the attributes of race and gender, four subgroups were derived. The four subgroups include white females, white males, non-white females, and non-white males. Descriptive statistics of baseline demographic characteristics were presented by gender and the baseline clinical characteristics by the race-gender classification. Multiple logistic regression was performed to determine potential correlates of the primary outcome variable—prescription of appropriate statin for the target population.

Five models were derived. Model I examines the race-gender relationship, while accounting for age rescales in SD units. For race-gender in the logistic model, white females were considered the reference group. Model II was Model I plus smoking status, with never smoked being the reference group. Model III=Model II plus hypertension status (with nonhypertension as reference group), A1C (SD units), and ASCVD risk score (SD units). Model IV is the fully adjusted model, which is Model III plus total cholesterol (SD units), LDL cholesterol (SD units), HDL cholesterol (SD units), and systolic blood pressure (SD units). Finally, Model V is the parsimonious model, which was derived using stepwise regression with a variable entry criterion of 0.05, and a variable exit criterion of 0.10.

## Results

In our study, there were 95 males and 97 females, with the average age of both genders being ∼55 years. The racial distributions of both genders were quite similar, with a slight difference in the proportion of black males and females. However, the smoking status of males and females is quite different, with 75.3% females never smoked compared to 50.5% males (Table 1). Overall, females (whites and non-whites) had lower A1C levels, higher HDL cholesterol, total cholesterol, lower percentage of ASCVD risk, and higher percentage of hypertension compared to their male counterparts (whites and non-whites) (Table 2). The biggest difference between males and females was in their calculated ASCVD risk.

**Table 1.** Baseline Demographic Characteristics of Study Participants

Characteristics	Male (*n*=95)	Female (*n*=97)	Total (*n*=192)
Age, in years; mean±SD (range)	55.3±(40–68)	50.5±(40–69)	55.5±7.2
Race, *n* (%)			
White	63 (66.3)	60 (61.9)	123 (64.1)
Non-white	32 (33.7)	37 (38.1)	69 (35.9)
Black/African American	15 (15.8)	24 (24.7)	39 (20.3)
Other	17 (17.9)	13 (13.4)	30 (15.6)
Smoking status, *n* (%)			
Current	16 (16.8)	12 (12.4)	28 (14.6)
Former	31 (32.6)	12 (12.4)	43 (22.4)
Never	48 (50.5)	73 (75.3)	121 (63.0)

SD, standard deviation.

**Table 2.** Baseline Clinical Characteristics of Study Participants by Gender-Race

Characteristics	Male			Female			Total sample (*n*=192)
	White (*n*=63)	Non-white (*n*=32)	Total (*n*=95)	White (*n*=60)	Non-white (*n*=37)	Total (*n*=97)	
Hemoglobin A1c	8.0±1.9	8.9±2.3	8.3±2.1	7.7±1.7	7.5±1.1	7.6±1.5	8.0±1.8
LDL cholesterol, mg/dL	112.0±26.1	114.0±31.0	112.7±27.7	115.4±28.5	111.8±29.9	114.0±28.9	113.4±28.3
HDL cholesterol, mg/dL	41.8±8.4	41.1±11.4	41.6±9.4	49.8±13.6	52.0±15.2	50.6±14.2	46.2±12.9
Total cholesterol, mg/dL	186.0±33.9	182.3±39.1	184.7±35.6	196.0±34.0	187.2±34.9	192.7±34.5	188.7±35.1
Systolic blood pressure, mmHg	131.5±18.1	138.3±16.5	133.8±17.8	131.5±20.2	132.1±15.4	131.8±18.4	132.8±18.1
ASCVD risk %	19.7±9.9	17.9±10.7	19.1±10.2	9.4±7.7	13.4±10.2	10.9±8.9	15.0±10.4
Documented hypertension treatment, *n* (%)	46 (73.0)	18 (53.3)	64 (67.4)	47 (78.3)	28 (75.7)	75 (77.3)	139 (72.4)
Documented statin prescription, *n* (%)	10 (15.9)	3 (9.4)	13 (13.7)	3 (5.0)	4 (10.8)	7 (7.4)	20 (10.4)
Intensity desired, *n* (%)^a^	45 (71.4)	21 (65.6)	66 (69.5)	44 (73.3)	26 (70.3)	70 (72.2)	136 (70.8)
Appropriate initial statin, yes; *n* (%)^a^	26 (57.8)	17 (81.0)	43 (65.2)	34 (77.3)	13 (50.0)	47 (67.1)	90 (66.2)

^a^ So, the row for “Intensity Desired” is counting those who got a statin prescription that's the exact intensity of what was desired. The next row for “Appropriate” counts those who got a desired intensity or higher (e.g., if someone was supposed to get a moderate intensity, but got a high intensity, it was counted here).

ASCVD, atherosclerotic cardiovascular disease; HDL, high-density lipoprotein; LDL, low-density lipoprotein.

The 10-year ASCVD risk in males was 19.10%, nearly double the 10.91% risk in females (*p*<0.001). Finally, in terms of statin therapy, our study actually found that females had higher rates of prescribed statins (72.16% vs. 69.47%) and received the appropriate intensity statin more often (48.45% vs. 45.26%), although neither of these rates were significant. For males, a high-intensity statin was desired significantly more often than for females (72.63% vs. 52.58%, *p*=0.0065). However, when looking at the actual statin intensities prescribed by gender, we found no significant difference across all intensities.

Table 3 provides results of a series of multivariable logistic regression examining the potential correlates to the use of appropriate statin therapy. The age-adjusted model (Model I) suggests that race-gender was a significant correlation with age not being a significant covariate. Using white females as a reference group, Model I suggested that non-white females were 71% less likely to be prescribed appropriate statin therapy compared to their white female counterparts. In Model II, after adjusting for age, which was significant in this case (odds ratio=0.62; 95% confidence interval: 0.40–0.98), and smoking status, which was not significant, it was found that non-white females were 71% less likely to be prescribed appropriate statin compared to their white female counterparts.

**Table 3.** Correlates of Prescription of Appropriate Statin to Patients with Diabetes Mellitus

Factors	Model I OR (95% CI)	Model II OR (95% CI)	Model III OR (95% CI)	Model IV OR (95% CI)	Model V OR (95% CI)
Age_SD (7.2 years)	0.66 (0.43–1.02)	0.62 (0.40–0.98)	0.79 (0.43–1.44)	0.83 (0.40–1.69)	—
Race-gender (Ref=White female)					
White male	0.44 (0.17–1.12)	0.45 (0.17–1.16)	0.57 (0.19–1.70)	0.66 (0.19–2.34)	0.68 (0.25–1.90)
Non-white female	0.29 (0.10–0.84)	0.29 (0.10–0.85)	0.35 (0.11–1.07)	0.40 (0.12–1.28)	0.36 (0.12–1.06)^a^
Non-white male	0.98 (0.26–3.6)	0.98 (0.25–3.82)	1.75 (0.35–8.90)	2.13 (0.36–12.53)	2.19 (0.53–8.98)^b^
Smoking status (Ref=never)					
Current	—	0.62 (0.21–1.85)	1.02 (0.24–4.27)	1.16 (0.23–5.99)	—
Former	—	1.11 (0.43–2.87)	1.25 (0.46–3.39)	1.28 (0.47–3.53)	—
Hypertension treatment (Ref=no)	—	—	0.46 (0.16–1.36)	0.42 (0.14–1.26)	—
A1c_SD (1.8%)	—	—	0.79 (0.51–1.22)	0.80 (0.50–1.26)	—
ASCVD_Risk_SD (10.4)	—	—	0.71 (0.39–1.32)	0.61 (0.23–1.63)	0.59 (0.39–0.89)
Total cholesterol_SD (35.1 mg/dL)	—	—	—	1.60 (0.58–4.38)	—
LDL cholesterol_SD (28.3 mg/dL)	—	—	—	0.64 (0.25–1.67)	—
HDL cholesterol_SD (12.9 mg/dL)	—	—	—	0.88 (0.54–1.43)	—
Systolic blood pressure_SD (18.1 mmHg)	—	—	—	1.19 (0.62–2.26)	—

—: No estimates due to factor not included in the model; Model I=Modeling the outcome variable with age (SD units); Model II=Model I plus smoking status; Model III=Model II plus hypertension status, A1C (SD units), and ASCVD risk score (SD units); Model IV=Model III plus total cholesterol (SD units), LDL cholesterol (SD units), HDL cholesterol (SD units), and systolic blood pressure (SD units); and Model V=parsimonious model (bases on stepwise regression).

^a^ Associated *p*=0.0165.

^b^ Associated *p*=0.0458.

CI, confidence interval; OR, odds ratio.

The significant association of race-gender with the use of appropriate statin therapy was attenuated in Models II (with the addition of hypertension status, A1C (SD units) and ASCVD risk score (SD units) as covariates. Similarly, the significant association between race-gender and the outcome variable (use appropriate statin therapy) was attenuated in the fully adjusted model, Model IV.

Hence, for the parsimonious model (Model V), the significant correlates selected by the program were race-gender and ASCVD Risk. ASCVD as a significant correlate suggests that as the ASCVD risk increased by 1 SD, the likelihood of being prescribed the appropriate statin for a person with diabetes decreases by 41%. It is also important to note, with the parsimonious model, non-white females are less likely, with non-white males being more likely, to be prescribed appropriate statin compared to white females.

## Discussion

In this cross-sectional study, about 30% of patients did not receive statin therapy for primary prevention. Females had a higher rate of prescribed statin therapy compared to males. Of those who did receive statin therapy, females were also more likely to receive the desired intensity based on the current 2018 ACC/AHA guidelines.^3^ These findings are contrary to several other studies that have identified gender disparities in statin therapy. Nanna et al. reported that women were significantly less likely to be on statin therapy compared to men (67.0% vs. 78.4%, respectively), as well as less likely to be on the guideline-recommended statin intensity (36.7% vs. 45.2%, respectively).^12^

It is difficult to determine why differences in prescribed statin therapy existed in our patients when comparing males to females, especially differences so far off from our original prediction. While males did have slightly higher average A1C and systolic blood pressure, females had slightly higher average total cholesterol. In addition, a greater percentage of females were on hypertension treatment, and females were less likely to be smokers. These may be contributing factors as to why there was such a large gap in their calculated ASCVD risk percentages, but do not explain why males were in fact the group with suboptimal treatment. One possibility that our study did not evaluate was the likelihood of which patients would be open to statin therapy.

Women may be more likely to seek out preventative measures as well as follow up with their primary care physicians. A study conducted by the Centers for Disease Control and Prevention (CDC) revealed that women with type 2 diabetes had a significantly greater rate of attending their clinic appointments.^14^ According to the National Health Interview Survey by the CDC, men were more likely to have had their last contact with a doctor or health care professional before 2 years when compared to women.^15^

Despite these trends, it is worth noting that males in our study were more likely to receive a higher intensity statin. A study conducted by Hammad et al. had found trends in statin therapy similar to this study.^16^ This study, similar to our study, had focused on type 2 diabetes and found that females were prescribed statin therapy more than males (83.2% compared to 78.9%).^16^ In their study, however, females had a higher average A1C of 8.5% compared to the 8.3% in males, a longer history of diabetes, and a greater family history of diabetes, which may all contribute to prescribers' rationale for statin therapy.^16^ Khan et al. reported that while males were more likely to be prescribed a statin, males were actually less likely to be on the recommended dosage compared to women.^17^

There was a substantial difference in the mean ASCVD risk score between males and females, with males having an average 10-year ASCVD risk of 19.10% compared to 10.91% in females. A previous study conducted by Morris et al. showed a similar trend with males having a higher 10-year ASCVD risk score compared to females. This trend demonstrating higher ASCVD risk scores in males was consistent among each race group, with males showing a higher average ASCVD risk score among whites, Hispanics, and blacks.^6^

The results of our study showed that a higher percentage of males compared to females were eligible for a high-intensity statin (72.63% compared to 52.58%, *p*=0.0065). Because of this, it was expected that a substantially higher number of males would be on a high-intensity statin compared to females; however, the study revealed no significant difference. This is clinically relevant because the 2019 ACC/AHA guidelines state that patients with higher ASCVD risk scores benefit more from statin therapy; however, patients in this study, who were shown to have a higher 10-year ASCVD risk, not only received less appropriate statin therapy but also received less statin therapy overall.^18^

In our study, only 10.42% of the patients had their ASCVD risk score documented by their providers, which could indicate as to why males did not receive the optimal statin therapy. This is an area of concern, considering the 2019 ACC/AHA Guidelines state that all patients who are being evaluated for cardiovascular disease primary prevention, should have their ASCVD score calculated before treatment.^18^ Not having an accessible calculated 10-year ASCVD risk score in these patients hinders the ability to make the appropriate treatment decisions.

The results in our study show that a larger percentage of white patients received initial statin therapy compared to non-white patients. These results are consistent with a former study conducted by Dorsch et al., which found race to be a significant predictor in statin therapy prescribing patterns.^19^ The study showed that within a cohort of patients with diabetes, blacks were less likely to receive a statin compared with white patients.^19^ When evaluating the data, it was initially hypothesized that our results would be in accordance with results from a study by Gamboa et al., in which white males received the most statins, followed by black males, white females, and then finally black females.^13^

In contrast, in our study, white females received the highest percentage of statins (73.3%), followed by white males (71.4%), then non-white females (70.3%), and finally non-white males (65.63%). Although these results were not similar to the results from Gamboa's study, it did demonstrate that white patients received a higher percentage of statin therapy compared to non-white patients, regardless of gender.

This disparity in statin therapy prescribing among black patients is clinically relevant due to the increased incidence of cardiovascular disease among black patients. According to a review, black patients who are disproportionately affected by cardiovascular disease have a higher burden of myocardial infarction, stroke, and heart failure compared to white patients.^20^

Similar to our study, a retrospective comparison of use of appropriate statin therapy conducted by Huff et al. also established the conclusion that appropriate statin therapy remains low among patients with diabetes.^21^ This study also demonstrated that males with diabetes were more likely to receive appropriate statin therapy when compared to females (53.1% vs. 26.3%, *p*=0.001). The gender differences in the Huff study, although are different from our study, still imply the same meaningful conclusion for the need of more appropriate statin prescribing patterns among patients with diabetes.^21^

### Health equity implications

To adequately assess the discrepancy in adherence to guideline recommendations and eliminate gender-race disparities, different strategies may be implemented. A survey for physicians may identify barriers specific to a clinic site. Perhaps another theme among physicians is the idea of “fire and forget.” This is a concept where ASCVD risk and LDL values might be calculated or obtained at a visit, but not reevaluated at subsequent visits. At a glance, providers are likely to see that they have prescribed a statin without considering that the recommendation for the intensity has changed.

A possible solution for this is suggesting to providers to reevaluate ASCVD scores and to obtain new LDL levels on a yearly basis to assess if the patient is on the optimal statin therapy. A Cochrane Collaboration Database's review highlights the significance of monitoring as an intervention to achieve compliance.^22^

Electronic medical record (EMR) reminders or flags for providers are currently used by EMR systems for laboratory values and recent procedures performed. This method will possibly allow real-time guideline recommendations, reduce the learning curve for the guidelines, and eliminate issues such as “fire and forget.”

A study conducted by Huff et al. explored the unique concept of pharmacists versus internal medicine teams managing statin therapy in patients with diabetes.^21^ The study did not find a significant difference in overall prescribed statins between those managed by a pharmacy team or an internal medicine team, but it did find a trend of more appropriate statin therapy being prescribed by pharmacists.^21^

Ultimately, none of these suggestions will have any improved clinical outcomes and eliminate gender-race disparities without providers and patients conforming to the importance of statin therapy. The buy-in can be achieved by eliciting concerns of providers, hospital administration, and patients. Only after concerns about the barriers to use of statin therapy have been reconciled can we expect EMR reminders, inclusion of pharmacists on health care teams, or the yearly assessments of LDL levels to be effective.

This retrospective, single-center study has limitations, including a small sample size, various prescribing patterns, and limited diversity. On the other hand, a strength of the study was that the data were collected over several years, which reflects larger study designs. Furthermore, it evaluated the use of statin therapy for primary prevention of cardiovascular events for a subgroup of patients with diabetes in a family medicine clinic.

## Conclusion

This study highlighted a gender disparity in statin prescribing that contraindicated previous studies. This study, in contrast, shows that although males had a significantly higher mean 10-year ASCVD risk score, they were less likely than their female counterparts to receive the appropriate-intensity statin therapy. Previous studies have shown race and gender disparities exist in the prevention of cardiovascular disease. A more collective, unified approach to improve prescribing patterns for statin therapy can eliminate these disparities. Further research and data analysis can reveal areas that may need the most attention.

## Abbreviations Used

ASCVD: atherosclerotic cardiovascular disease
CARDS: Collaborative Atorvastatin Diabetes Study
CDC: Centers for Disease Control and Prevention
CI: confidence interval
EMR: electronic medical record
HDL: high-density lipoprotein
ICD: International Classification of Diseases
JAHA: *Journal of American Heart Association*
LDL: low-density lipoprotein
OR: odds ratio
SDs: standard deviations
